# Prevalence, regional distribution, and trends of antimicrobial resistance among female outpatients with urine *Klebsiella* spp. isolates: a multicenter evaluation in the United States between 2011 and 2019

**DOI:** 10.1186/s13756-024-01372-x

**Published:** 2024-02-14

**Authors:** Keith S. Kaye, Vikas Gupta, Aruni Mulgirigama, Ashish V. Joshi, Gang Ye, Nicole E. Scangarella-Oman, Kalvin Yu, Fanny S. Mitrani-Gold

**Affiliations:** 1 Division of Allergy, Immunology and Infectious Diseases, Rutgers – Robert Wood Johnson Medical School, New Brunswick, NJ USA; 2 MMS Medical Affairs, Becton, Dickinson and Company, Franklin Lakes, NJ USA; 3 Antibiotics, GSK, Brentford, London, UK; 4 Value Evidence and Outcomes, GSK, Collegeville, PA USA; 5 Software Technology Solutions, Becton, Dickinson and Company, Franklin Lakes, NJ USA; 6 Diagnostics and Clinical Microbiology, GSK, Collegeville, PA USA; 7 Medical and Scientific Affairs, Becton, Dickinson and Company, Franklin Lakes, NJ USA; 8 Epidemiology, GSK, Collegeville, PA USA

**Keywords:** *Klebsiella pneumoniae*, *Klebsiella oxytoca*, Antimicrobial resistance, Uncomplicated urinary tract infection

## Abstract

**Background:**

Antimicrobial resistance research in uncomplicated urinary tract infection typically focuses on the main causative pathogen, *Escherichia coli*; however, little is known about the antimicrobial resistance burden of *Klebsiella* species, which can also cause uncomplicated urinary tract infections. This retrospective cohort study assessed the prevalence and geographic distribution of antimicrobial resistance among *Klebsiella* species and antimicrobial resistance trends for *K. pneumoniae* in the United States (2011–2019).

**Methods:**

*K. pneumoniae* and *K. oxytoca* urine isolates (30-day, non-duplicate) among female outpatients (aged ≥ 12 years) with presumed uUTI at 304 centers in the United States were classified by resistance phenotype(s): not susceptible to nitrofurantoin, trimethoprim/sulfamethoxazole, or fluoroquinolone, extended-spectrum β-lactamase-positive/not susceptible; and multidrug-resistant based on ≥ 2 and ≥ 3 resistance phenotypes. Antimicrobial resistance prevalence by census division and age, as well as antimicrobial resistance trends over time for *Klebsiella* species, were assessed using generalized estimating equations.

**Results:**

270,552 *Klebsiella* species isolates were evaluated (250,719 *K. pneumoniae*; 19,833 *K. oxytoca*). The most frequent resistance phenotypes in 2019 were nitrofurantoin not susceptible (*Klebsiella* species: 54.0%; *K. pneumoniae*: 57.3%; *K. oxytoca*: 15.1%) and trimethoprim/sulfamethoxazole not susceptible (*Klebsiella* species: 10.4%; *K. pneumoniae*: 10.6%; *K. oxytoca*: 8.6%). Extended-spectrum β-lactamase-positive/not susceptible prevalence was 5.4%, 5.3%, and 6.8%, respectively. *K. pneumoniae* resistance phenotype prevalence varied (*p* < 0.0001) geographically and by age, and increased over time (except for the nitrofurantoin not susceptible phenotype, which was stable and > 50% throughout).

**Conclusions:**

There is a high antimicrobial resistance prevalence and increasing antimicrobial resistance trends among *K. pneumoniae* isolates from female outpatients in the United States with presumed uncomplicated urinary tract infection. Awareness of *K. pneumoniae* antimicrobial resistance helps to optimize empiric uncomplicated urinary tract infection treatment.

**Supplementary Information:**

The online version contains supplementary material available at 10.1186/s13756-024-01372-x.

## Introduction

Antimicrobial resistance (AMR) is a serious health threat [1–3]. Urinary tract infections (UTIs) are among the most common bacterial infections in the United States (US), occurring in ~ 11% of adult females each year [4]. Most community-acquired UTIs in female patients are uncomplicated UTIs (uUTIs), meaning they occur in patients with no structural or functional abnormalities of the urinary tract, nor any complicating comorbidities (uncontrolled/complicated diabetes, immunosuppression, or pregnancy) [4, 5]. AMR is increasing, making the management of uUTIs more challenging [6–11].

*Escherichia coli* is the most common cause of community-acquired uUTIs, and previous work has determined the prevalence and geographic distribution of AMR among *E. coli* in the US [9, 12]. One such study demonstrated a high prevalence of non-susceptibility to trimethoprim/sulfamethoxazole (SXT; 25.4%) and fluoroquinolones (FQs; 21.1%) among *E. coli* isolates [12]. Further, the study showed an increasing trend of extended-spectrum β-lactamase (ESBL)-producing *E. coli* isolates over recent years, with relative average yearly increases of 7.7% (95% confidence interval [CI], 7.2–8.2%; *p* < 0.0001) [12]. However, other bacteria are the causative agents in ~ 25% of uUTIs with *Klebsiella pneumoniae* alone causing approximately 6% of uUTIs [13]. There has been a notable increase in AMR among *K. pneumoniae* in the US and elsewhere in recent years, and issues related to multidrug resistance (MDR) have been highlighted in the literature [9, 14, 15]. Resistance mechanisms in *K. pneumoniae* can include the production of ESBLs, plasmid-encoded AmpC β-lactamases, and class A, B, and D carbapenemases [14, 16].

Urine cultures are not routinely obtained for the diagnosis of uUTI and treatment is primarily empiric; however, urine cultures are increasingly recommended due to growing resistance rates [4, 6, 8]. Despite this, European and US data show that urine cultures are only performed in approximately 17% of patients with UTIs, and only 7% of those are at a low risk of complications (low-risk patients have no high-risk features such as male sex, immune disorder, or pregnancy) [17, 18]. Therefore, regional outpatient AMR surveillance data are often limited to patients with complicated UTI and may not be representative of community-acquired uUTI [9, 10, 19]. Availability of contemporary AMR surveillance data on outpatient isolates is important to enable treating physicians to provide optimal empiric antimicrobial treatment for patients [19]. This study was designed to assess the prevalence and geographic distribution of AMR among *Klebsiella* spp. uropathogens (*K. pneumoniae* and *K. oxytoca*) and to assess AMR trends among *K. pneumoniae* urine isolates from female outpatients in the US.

## Methods

### Study design

**Fig. 1:**
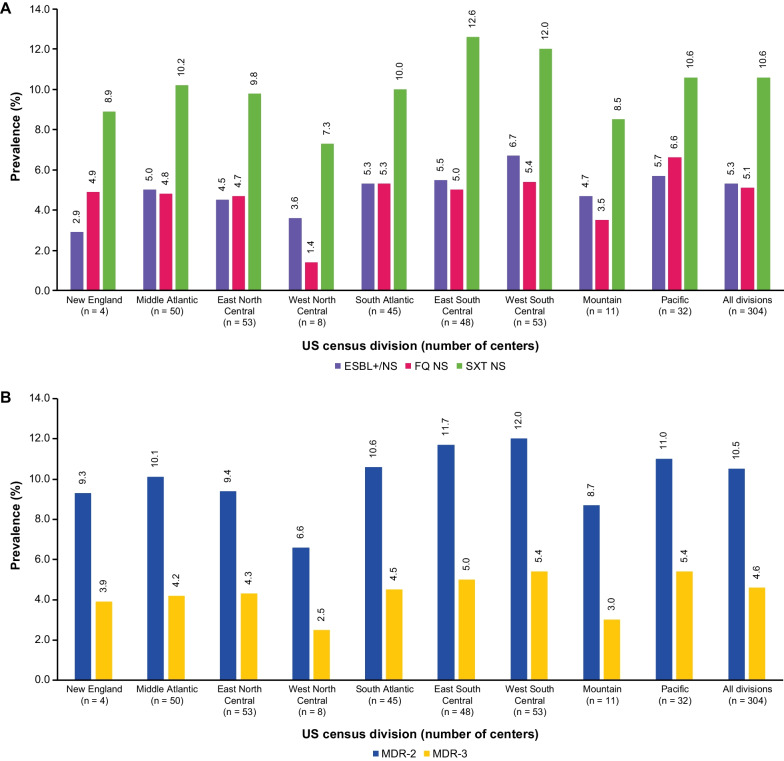
Prevalence of AMR among 30-day non-duplicate *K. pneumoniae* isolates in 2019 by US census division. For panel **A**, data are shown for ESBL+/NS, FQ NS, SXT NS; NTF NS are not depicted to provide greater clarity for the three other AMR phenotypes. By US census division, the NTF NS prevalence was 53.3% in New England, 58.2% in the Middle Atlantic, 56.6% in East North Central, 54.5% in West North Central, 60.4% in the South Atlantic, 57.6% in East South Central, 56.0% in West South Central, 53.8% in Mountain, and 56.3% in the Pacific. New England: CT, MA, ME, NH, RI, VT; Middle Atlantic: NJ, NY, PA; East North Central: IL, IN, MI, OH, WI; West North Central: IA, KS, MN, MO, ND, NE, SD; South Atlantic: DE, DC, FL, GA, MD, NC, SC, VA, WV; East South Central: AL, KY, MS, TN; West South Central: AR, LA, OK, TX; Mountain: AZ, CO, ID, MT, NM, NV, UT, WY; Pacific: AK, CA, OR, WA. AMR, Antimicrobial resistance; ESBL+/NS, Extended spectrum β-lactamase-producing or not susceptible to ceftriaxone, cefotaxime, ceftazidime, or cefepime; FQ, Fluoroquinolone; MDR-2/-3, Multidrug-resistant if resistant to ≥ 1 antibiotic in ≥ 2 or ≥ 3 drug classes (including NTF, SXT, FQ, or the ESBL+/NS phenotype); NS, Not susceptible; NTF, Nitrofurantoin; SXT, Trimethoprim/sulfamethoxazole; US, United States

This was a retrospective, multicenter, cohort study of *Klebsiella* spp. (*K. pneumoniae* and *K. oxytoca*) urine isolates identified from female patients seeking care in an outpatient/ambulatory healthcare setting (e.g., emergency department, physician’s office, ambulatory clinic) aged ≥ 12 years with presumed uUTI, a positive urine culture (non-contaminant isolate with a microbiological work-up based on adequate colony count per local reference lab practice) and no subsequent admission in the next 24 h. The study was conducted in compliance with Health Insurance Portability and Accountability Act requirements. Outpatient urine isolates were collected from 304 US hospital facilities included in the BD Insights Research Database (Becton, Dickinson and Company, Franklin Lakes, NJ, US) between January 2011 and December 2019. The distribution of hospitals in the database by US census divisions is shown in Fig. 1 and comprises small and large hospitals in urban and rural locations [2].

Eligible isolates were 30-day non-duplicate, defined as the first urine *Klebsiella* spp. per patient collected within 30 days. Subsequent *Klebsiella* spp. isolates from the same patient were included if collected > 30 days from the previous isolate. If additional urine *Klebsiella* spp. isolates of the same genus and species from the same patient were recovered within 30 days, they were only included if they had different drug susceptibilities (at least one interpretive criteria difference). Data identified included: study year, age group, US census division, and isolated *Klebsiella* spp. uropathogen(s); tested antibiotics; and healthcare facility type, size, and geographic location. Regional AMR prevalence (by US census division) in 2019 was assessed among all *Klebsiella* spp. isolates and trends in AMR between 2011 and 2019 among *K. pneumoniae* isolates only.

The study was performed using a de-identified limited retrospective dataset that was deemed exempt from patient consent by the New England Institutional Review Board/Human Subjects Research Committee (Wellesley, MA, US).

### Determination of AMR phenotypes

*Klebsiella* spp. urine isolates were classified into the following six microbiological AMR phenotypes: not susceptible (intermediate/resistant; NS) to (1) nitrofurantoin (NTF), (2) SXT, or (3) FQ; (4) ESBL-positive (ESBL+)/NS, confirmed by a commercial panel or NS to ceftriaxone, cefotaxime, ceftazidime, or cefepime; (5) multidrug-resistant to ≥ 1 drug in ≥ 2 drug classes (MDR-2) or (6) multidrug-resistant to ≥ 1 drug in ≥ 3 drug classes (MDR-3). The drug classes considered for MDR classifications included all possible combinations of all four aforementioned AMR phenotypes. For comparison with the most current year of data (2019), AMR data for *Klebsiella* spp. were evaluated for each year between 2011 and 2018, including prevalence of the phenotypes described above and with stratification for the genus overall (*Klebsiella* spp.) and by species (*K. pneumoniae* and *K. oxytoca*). Individual laboratories at each institution performed the antimicrobial susceptibility testing using Clinical and Laboratory Standards Institute (CLSI)-approved methods and interpreted results according to Food and Drug Administration (FDA)/CLSI breakpoints and interpretative criteria [20]. The term NS is used per the CLSI guidelines definition of NS in this manuscript (the term “non-susceptible” is the counterpart in American Medical Association guidance); NS and AMR are also used interchangeably.

### Statistical analysis

AMR prevalence was calculated as a percentage (NS isolates by genus/species divided by total isolates tested by genus/species × 100) for *Klebsiella* spp. overall and for *K. pneumoniae* and *K. oxytoca* for the entire study period (2011–2019) by study year, US census division, and categorical age group, for each resistance phenotype (NTF NS, SXT NS, FQ NS, ESBL+, MDR-2, or MDR-3). Bivariate analyses using logistic regression models were performed to test for statistical differences in AMR prevalence by study year, age group, US census divisions, and other facility characteristics (bed size, teaching status, and urban *vs.* rural status). Final estimates for AMR prevalence and resistance trends (2011–2019) for *K. pneumoniae* were assessed using generalized estimating equations (GEE). Mean relative annual percent change in AMR between 2011 and 2019 were estimated for each of the six resistance phenotypes using the GEE models. *P*-values were generated for comparisons of AMR for each resistance phenotype by US census division and age group; statistical significance was defined at the 5% level. All analyses were conducted using the Statistical Analysis System (SAS) V9.4 (SAS Institute, Cary, NC, USA).

## Results

The full study cohort (2011–2019) comprised 270,552 non-duplicate *Klebsiella* spp. isolates; of these, 250,719 (92.7%) were *K. pneumoniae* and 19,833 (7.3%) were *K. oxytoca*.

### Descriptive analysis: prevalence of AMR in 2019

The total number of *Klebsiella* spp. isolates in 2019 was 47,755; comprised of 44,056 (92.3%) *K. pneumoniae* and 3699 (7.7%) *K. oxytoca*. Of the most common AMR phenotypes, the overall prevalence of *Klebsiella* spp. NS to NTF, SXT, and with MDR-2 in 2019 was 54.0%, 10.4%, and 10.2%, respectively. The most common AMR phenotype was NTF NS (*K. pneumoniae*: 57.3%; *K. oxytoca*: 15.1%). The prevalence of the ESBL+/NS phenotype was 5.4% for *Klebsiella* spp., comprising 5.3% for *K. pneumoniae* and 6.8% for *K. oxytoca*.

The prevalence of each AMR phenotype in 2019 varied between US census divisions. For *Klebsiella* spp., the prevalence of the AMR phenotypes across all divisions ranged from 48.2–58.5% for NTF NS, 6.9–12.3% for SXT NS, 6.1–12.0% for MDR-2, 3.2–7.2% for ESBL+/NS, 1.4–6.6% for FQ NS, and 2.1–5.4% for MDR-3. Equivalent prevalence data are described in Fig. 1 for *K. pneumoniae* and Additional file 1: Fig. S1 for *K. oxytoca.*

For *K. pneumoniae*, prevalence of AMR phenotypes by US census divisions are summarized in Fig. 1A and B. All divisions had NTF NS prevalence > 50%, but the highest prevalence was observed in the South Atlantic (60.4%) and Middle Atlantic (58.2%) divisions. SXT NS prevalence ranged from 7.3% in the West North Central division to 12.6% in the East South Central division. ESBL+/NS prevalence ranged from 2.9% in the New England division to 6.7% in the West South Central division. FQ NS prevalence ranged from 1.4% in the West North Central division to 6.6% in the Pacific division. MDR-2 and MDR-3 prevalence was highest in the West South Central division (12.0% and 5.4%, respectively; Fig. 1B).

For *K. oxytoca*, the highest prevalence of NTF NS in 2019 was observed in the Mountain (19.8%) division, while the lowest prevalence was observed in the New England (7.7%) division (see Additional file 1: Fig. S1A). SXT NS prevalence ranged from 4.3% in the Middle Atlantic division to 16.3% in the West South Central division. ESBL+/NS prevalence ranged from 2.8% in the West North Central division to 13.5% in the West South Central division. FQ NS prevalence ranged from 0.0% in the New England division to 7.0% in the West South Central division. MDR-2 and MDR-3 prevalence was highest in the West South Central division (12.1% and 4.9%, respectively) and lowest in the New England division (1.5% and 0.0%, respectively); the MDR-3 prevalence was 0.0% in the West North Central division (see Additional file 1: Fig. S1B).

### Descriptive trend analysis: AMR prevalence among *Klebsiella* spp. (2011–2019)

**Fig. 2:**
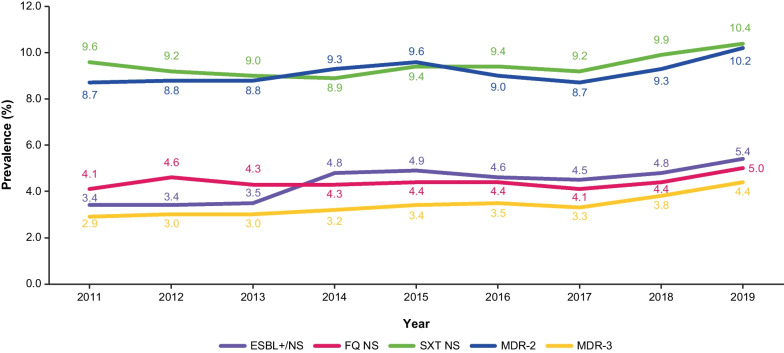
Observed AMR prevalence trends among 30-day non-duplicate *Klebsiella* spp. isolates from US female outpatients. Data for NTF NS are not depicted to provide greater clarity for the five other AMR phenotypes (NTF NS prevalence was > 50.0% throughout; these data can be found in Additional file 1: Fig. S2). AMR, Antimicrobial resistance; ESBL+/NS, Extended spectrum β-lactamase-producing or not susceptible to ceftriaxone, cefotaxime, ceftazidime, or cefepime; FQ, Fluoroquinolone; MDR-2/-3, Multidrug-resistant if resistant to ≥ 1 antibiotic in ≥ 2 or ≥ 3 drug classes (including NTF, SXT, FQ, or the ESBL+/NS phenotype); NS, not susceptible; NTF, Nitrofurantoin; SXT, Trimethoprim-sulfamethoxazole; US, United States

Trends in AMR prevalence for each phenotype by year over the study period (2011–2019) are shown in Fig. 2. Increases were observed for all of the phenotypes except NTF NS. NTF NS prevalence data are presented in Additional file 1: Fig. S2.

### Modeled analysis: AMR trends among *K. pneumoniae* isolates (2011–2019)

The GEE model-estimated prevalence of each AMR phenotype among the 250,719 *K. pneumoniae* isolates evaluated is shown in Table 1. After adjusting for age group, US census division, and other facility characteristics (bed size, teaching status, and urban *vs.* rural status), the overall prevalence of AMR ranged from 3.7% for MDR-3 to 56.6% for NTF NS across the 2011–2019 study period. There was significant variation in adjusted AMR phenotype prevalence among *K. pneumoniae* isolates between US census divisions (*p* < 0.0001 for all phenotypes except NTF NS [*p* < 0.005]). For NTF NS, the highest prevalence was observed in the South Atlantic (61.6%) and Pacific (58.6%) divisions, with all other divisions having an NTF NS prevalence of > 50%. The West North Central and New England divisions tended to show the lowest prevalence for ESBL+, FQ NS, and SXT NS in general. MDR-2 and MDR-3 prevalences were highest in the West South Central (12.2% and 4.3%, respectively) and Pacific (11.7% and 4.0%, respectively) divisions, and the lowest MDR-2 and MDR-3 prevalence was observed in the West North Central (8.1% and 2.3%, respectively) and New England (8.0% and 2.8%, respectively) divisions. AMR phenotypes also differed significantly between age groups from 2011–2019 (*p* < 0.0001; Table 1). The prevalence of NTF NS decreased with increasing age (from 61.4% at 12–17 years to 52.2% at > 74 years), while the other phenotypes were more prevalent among patients in age groups ≥ 55 years than those aged < 55 years (Table 1).

**Table 1:** Model-estimated AMR prevalence of *K. pneumoniae* isolates from female outpatients with presumptive uUTI (2011–2019)

AMR phenotype prevalence, % (95% Cl)						
Characteristic	NTF NS (n = 141,799)	SXT NS (n = 24,027)	ESBL+/NS (n = 11,181)	FQ NS (n = 11,075)	MDR-2	MDR-3
Overall prevalence (2011–2019)	56.6 (56.3–56.9)	9.6 (9.5–9.7)	4.6 (4.5–4.7)	4.4 (4.3–4.5)	9.5 (9.3–9.6)	3.7 (3.6–3.8)
Average annual percent change (2011–2019)^a^	− 0.3 (−0.6 to −0.1)	+ 2.1 (1.6 to 2.7)	+ 5.4 (4.3 to 6.5)	+ 2.1 (1.1 to 3.0)	+ 1.8 (1.1 to 2.4)	+ 5.6 (4.5 to 6.8)
Age (years)^b^						
12–17	61.4 (58.8–64.1)	8.3 (7.3–9.4)	1.9 (1.5–2.4)	1.4 (1.1–1.9)	7.1 (6.2–8.2)	1.5 (1.1–2.0)
18–54	59.1 (58.4–59.9)	8.9 (8.2–9.7)	3.0 (2.8–3.2)	2.9 (2.5–3.4)	8.7 (8.0–9.5)	2.6 (2.3–3.1)
55–64	57.4 (56.5–58.4)	11.6 (10.7–12.6)	4.7 (4.3–5.0)	5.3 (4.6–6.2)	11.7 (10.7–12.8)	4.8 (4.1–5.6)
65–74	55.0 (54.2–55.8)	11.2 (10.4–12.2)	4.4 (4.2–4.7)	5.2 (4.5–6.1)	11.3 (10.4–12.4)	4.6 (4.0–5.4)
> 74	52.2 (51.6–52.9)	10.2 (9.4–11.0)	4.4 (4.2–4.7)	5.5 (4.8–6.4)	10.7 (9.8–11.6)	4.5 (3.9–5.3)
US census division^b^						
New England	57.7 (55.0–60.5)	9.0 (7.8–10.4)	2.5 (2.0–3.2)	2.8 (2.2–3.6)	8.0 (6.8–9.3)	2.8 (2.1–3.6)
Middle Atlantic	54.4 (53.5–55.3)	9.8 (9.1–10.6)	3.4 (3.1–3.6)	3.8 (3.3–4.5)	9.1 (8.4–9.9)	3.4 (2.9–3.9)
East North Central	56.2 (55.4–57.0)	9.8 (9.1–10.6)	3.2 (3.0–3.4)	3.7 (3.2–4.3)	9.2 (8.5–10.0)	3.4 (2.9–3.9)
West North Central	56.0 (53.2–59.0)	8.6 (7.4–10.1)	2.5 (1.9–3.2)	2.4 (1.8–3.1)	8.1 (6.9–9.6)	2.3 (1.7–3.1)
South Atlantic	61.6 (60.6–62.6)	10.2 (9.4–11.1)	3.9 (3.6–4.3)	4.4 (3.7–5.1)	11.0 (10.1–12.0)	3.8 (3.3–4.5)
East South Central	55.5 (54.6–56.5)	11.4 (10.6–12.3)	3.5 (3.2–3.7)	3.8 (3.3–4.5)	10.4 (9.5–11.2)	3.3 (2.8–3.8)
West South Central	56.0 (55.1–56.9)	12.1 (11.2–13.1)	4.9 (4.5–5.3)	4.6 (3.9–5.3)	12.2 (11.3–13.2)	4.3 (3.7–5.0)
Mountain	56.8 (55.2–58.5)	8.9 (8.0–9.8)	3.1 (2.7–3.6)	3.6 (3.0–4.3)	8.8 (7.9–9.8)	3.0 (2.5–3.6)
Pacific	58.6 (57.5–59.7)	10.3 (9.5–11.2)	5.4 (5.0–5.9)	4.4 (3.7–5.1)	11.7 (10.7–12.8)	4.0 (3.4–4.7)

AMR, Antimicrobial resistance; CI, Confidence interval; ESBL+/NS, Extended-spectrum β-lactamase-positive or not susceptible to ceftriaxone, cefotaxime, ceftazidime, or cefepime; FQ, Fluoroquinolone; MDR-2/-3, Multidrug-resistant if resistant to ≥ 1 antibiotic in ≥ 2 or ≥ 3 individual drug classes (including NTF, SXT, FQ, or the ESBL+/NS phenotype); NS, Not susceptible; NTF, Nitrofurantoin; SXT, Trimethoprim/sulfamethoxazole; US, United States; uUTI, Uncomplicated urinary tract infection

^a^Relative average annual percentage change in prevalence of resistance. NTF NS *p* = 0.0041; all other phenotypes *p* < 0.0001

^b^Significant variation observed across age groups (*p* < 0.0001) and census divisions (*p* < 0.0001) for all phenotypes

The assessment of trends in AMR prevalence using statistical models (GEE models) showed that the estimated relative annual percent change in AMR prevalence was highest for MDR-3 and ESBL+/NS (+ 5.6% and + 5.4%, respectively) and lowest for MDR-2 and NTF NS (+ 1.8% and − 0.3%, respectively) (*p* < 0.0001 for all phenotypes except NTF NS [*p* < 0.005]) (Table 1).

## Discussion

The prevalence of AMR in outpatient *Klebsiella* spp. urine isolates was high and increased for most phenotypes throughout the study period; NTF NS prevalence did not increase but were as high as 54.0% for *Klebsiella* spp. and 57.3% for *K. pneumoniae* in 2019. Overall MDR-2 prevalence exceeded 10% for *Klebsiella* spp. and *K. pneumoniae*. Equivalent prevalence in 2019 was markedly lower for *K. oxytoca*, which demonstrated a NTF NS prevalence of 15.1% and MDR-2 prevalence of 6.5%. The prevalence of the ESBL+/NS phenotype in 2019, however, was higher for *K. oxytoca* isolates (6.8%) compared with *K. pneumoniae* isolates (5.3%).

AMR prevalence for *K. pneumoniae* differed significantly between US census divisions. Some divisions had relatively high AMR prevalence for multiple phenotypes, including the South Atlantic, West South Central, and East South Central divisions. This finding supports previous observations of high AMR prevalence in US southern-border regions, which may be influenced by access to antimicrobials, and their overuse, without a prescription in Mexican pharmacies along the US-Mexico border [21]. Studies have also demonstrated higher outpatient antibiotic prescribing, including inappropriate prescribing, in Southern US states versus other US regions [1, 22], which might reflect regional differences in antibiotic stewardship [1, 22].

AMR prevalence for *K. pneumoniae* also differed between age groups. The prevalence of AMR was higher among females aged ≥ 55 years for most of the AMR phenotypes; however, prevalence of the NTF NS phenotype decreased as age increased. It is noteworthy that the 2015 American Geriatrics Society recommendations were not supportive of NTF use in elderly patients with renal insufficiency and low creatinine clearance [23].

The rapid spread of pathogens with the ESBL+ phenotype is of global concern, as effective empiric oral therapeutic options are limited and the burden of AMR in acute care settings is increasing. In our study, the model-estimated overall prevalence of ESBL+/NS *K. pneumoniae* isolates over the 2011–2019 study period was 4.6% (across all census divisions), with a relative annual increase in prevalence of 5.4% over this same period. These data are comparable to previous studies of ESBL+ prevalence in the US [9, 24–26]. The estimated relative annual increase in MDR-3 prevalence (+ 5.6%) that we observed was marginally above that for ESBL+/NS (+ 5.4%) and indeed the two may be correlated. NTF NS was the only AMR phenotype for which the estimated prevalence did not increase between 2011 and 2019; this may be partly due to the aforementioned American Geriatrics Society recommendations, which were published in 2015 [23]. However, despite the decrease in NTF NS prevalence during the study period, the overall prevalence of NTF NS was high (56.6%; NTF NS prevalence was > 50% for each year, and > 50% across all census divisions in 2019), highlighting a reduced likelihood of clinical efficacy for NTF when used empirically to treat community-acquired UTIs presenting to the emergency department. This is important to consider when updating clinical practice guidelines for the treatment of uUTI, such as those published by the Infectious Diseases Society of America (IDSA) in 2011, which recommend NTF as a first-line treatment for uUTI [27].

The prevalence of AMR phenotypes among community *K. pneumoniae* urine isolates in the US has not previously been well characterized, although such studies have been conducted in other countries, including Spain and China [14, 28, 29]. US surveillance of samples from inpatients (clinical setting not specified; taken between 1998–2010) showed that *K. pneumoniae* AMR prevalence has increased over time, reaching 19.3% for SXT and 16.8% for ciprofloxacin in 2010 [30]. Our study expands on these findings for the years 2011 and beyond, with a focus on outpatients in the US. Over the 2011–2019 period evaluated herein, the estimated overall prevalence of the SXT NS and FQ NS phenotypes among *K. pneumoniae* isolates was 9.6% and 4.4%, respectively, with a relative annual increase in prevalence of 2.1% for both phenotypes. Due to potentially serious side effects and the availability of other effective antibiotics as preferred first-line agents (e.g., NTF, SXT), guidelines recommend that FQs are reserved for patients who have no other options for the treatment of uUTI [27].

This study describes a large sample of *K. pneumoniae* isolates from US outpatients over nine years and provides valuable insights into prevalence of AMR among urine isolates in this setting. Electronic outpatient microbiology results were available for all eligible patients, which provided a comprehensive data source for the evaluation of AMR among *Klebsiella* spp. uropathogens.

Limitations of the study include potential variability in susceptibility testing due to reliance on local laboratory practices. The lack of information on specific laboratory practices within census divisions also precludes more localized resistance data. The study period included various changes in the minimum inhibitory concentration breakpoints and interpretive criteria from the CLSI for cefazolin, cefepime, levofloxacin, and ciprofloxacin; when these changes were implemented by individual laboratories involved in the study is unknown, which may have led to inconsistencies between centers over time [20]. The patient isolates used in the study could not definitely be linked to a uUTI diagnosis (via International Classification of Diseases-9/10 diagnosis codes), clinical symptoms, or pharmacy claims for antimicrobial prescribing. The potential inclusion in our study of outpatients with recurrent uUTI (due to the inclusion of more than one sample from the same patient with an interval exceeding 30 days) could have potentially overestimated the prevalence of AMR. Similarly, selection bias may have led to the inclusion of more resistant isolates due to the inclusion of isolates for patients with uUTI from whom a specimen was collected and analyzed (as opposed to any patient with uUTI). Although the data provide coverage of the US, the distribution of participating centers at a regional level may under-represent certain areas and specific patient subgroups, and results may not be generalizable to the entire female US population. The study was designed to include isolates from outpatients without a hospitalization within 24 h of urine culture; however, the possibility of hospitalization to a hospital not affiliated with the BD Insights Research Database could not be ruled out. Finally, the prevalence of AMR to fosfomycin was not assessed due to its limited use in the US, the requirement of a laborious reference standard method (agar dilution) that is not commonly used in diagnostic microbiology [31], and its omission from commercial susceptibility panels in the US.

## Conclusion

Awareness of AMR patterns among outpatient *Klebsiella* spp. uropathogens, particularly *K. pneumoniae*, is crucial to help guide physicians in the optimal empiric treatment of uUTI. This large study confirms high NTF NS and increasing AMR trends among urinary *K. pneumoniae* and *K. oxytoca* isolates from female outpatients in the US. Prevalence of resistance was highest for NTF and the highest estimated relative annual AMR increases among *K. pneumoniae* were observed for the ESBL+ (5.4%) and MDR-3 (5.6%) phenotypes. The prevalence of AMR differed significantly between census divisions within the US and by outpatient age group. These surveillance findings can be used to inform empiric prescribing for uUTI in the community setting. Ongoing surveillance of community isolates is necessary to ensure that female patients continue to receive effective empiric antibiotic therapy for uUTI.

### Supplementary Information

**Additional file 1: Figure S1.** Prevalence of AMR among 30-day non-duplicate *K. oxytoca* isolates in 2019 by US census division: **A** ESBL+/NS, NTF NS, FQ NS, and SXT NS and **B** MDR-2 and MDR-3. **Figure S2.** Observed NTF NS prevalence trends among 30-day non-duplicate *Klebsiella* spp. isolates from female outpatients in the US over the 2011–2019 study period.

## Data Availability

The authors confirm that the data supporting the findings of this study are available within the article and supplementary information.

## Abbreviations

AMR: Antimicrobial resistance
CLSI: Clinical and Laboratory Standards Institute
ESBL: Extended-spectrum β-lactamase
ESBL+: Extended-spectrum β-lactamase-positive
*E. coli*: *Escherichia coli*
FDA: Food and Drug Administration
FQ: Fluoroquinolone
GEE: Generalized estimating equations
*K. oxytoca*: *Klebsiella oxytoca*
*K. pneomoniae*: *Klebsiella pneumoniae*
MDR: Multidrug resistance
NS: Not susceptible
NTF: Nitrofurantoin
SAS: Statistical Analysis System
spp.: Species
SXT: Trimethoprim/sulfamethoxazole
US: United States
UTI: Urinary tract infection
uUTI: Uncomplicated urinary tract infection
