# Supplementary material for: Prevalence, regional distribution, and trends of antimicrobial resistance among female outpatients with urine Klebsiella spp. isolates: a multicenter evaluation in the United States between 2011 and 2019
Source: Antimicrob Resist Infect Control. 2024 Feb 14;13:21. doi: 10.1186/s13756-024-01372-x (PMC10865585; doi:10.1186/s13756-024-01372-x)
Supplement: Supplementary file 1 — Additional file 1: Figure S1. Prevalence of AMR among 30-day non-duplicate K. oxytoca isolates in 2019 by US census division: A ESBL+/NS, NTF NS, FQ NS, and SXT NS and B MDR-2 and MDR-3. Figure S2. Observed NTF NS prevalence trends among 30-day non-duplicate Klebsiella spp. isolates from female outpatients in the US over the 2011–2019 study period. [file 13756_2024_1372_MOESM1_ESM.docx]

**Supplementary Figures**

**Supplementary Fig. 1** Prevalence of AMR among 30-day non-duplicate *K. oxytoca* isolates in 2019 by US census division: (A) ESBL+/NS, NTF NS, FQ NS, and SXT NS and (B) MDR-2 and MDR-3


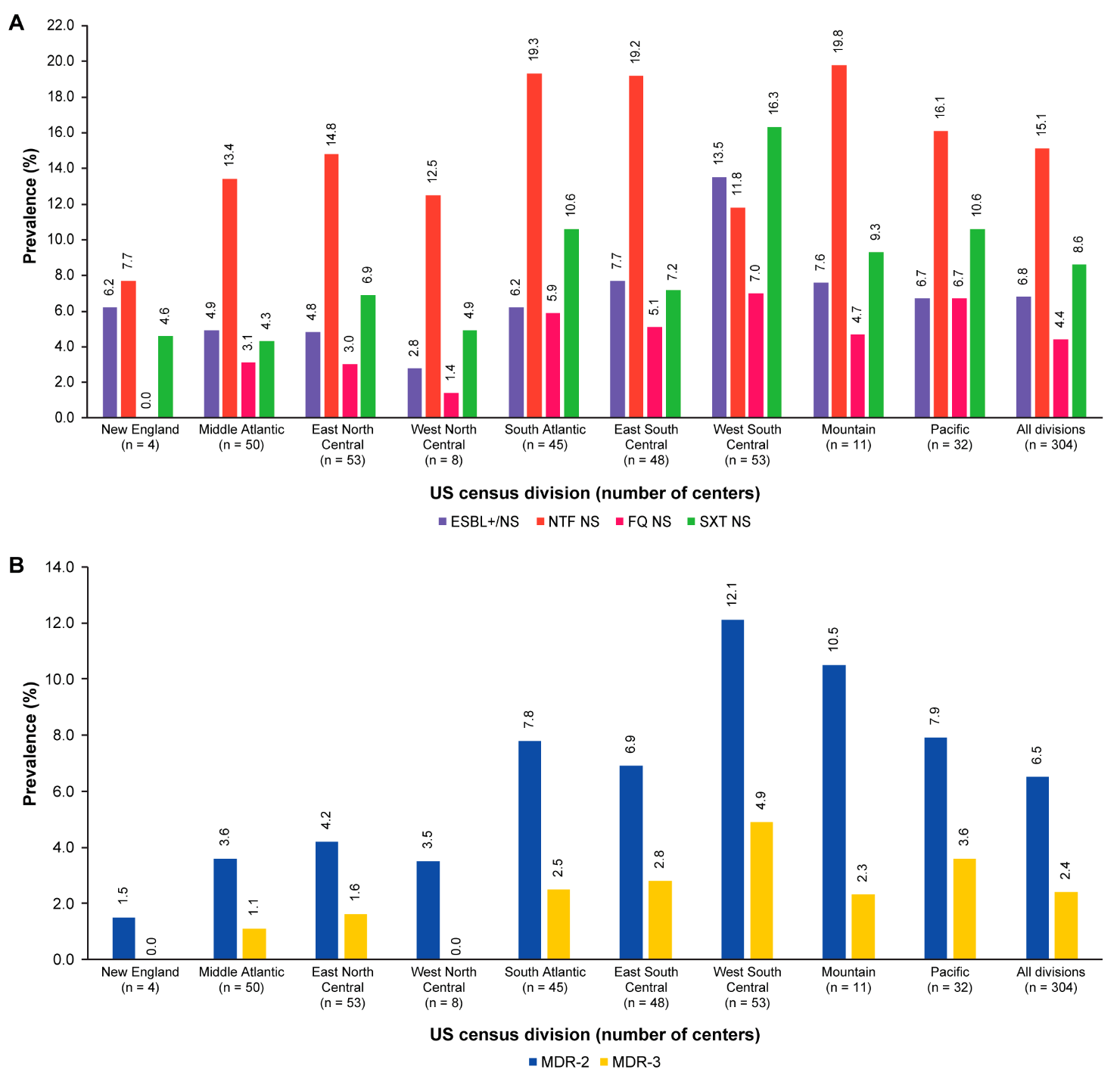


AMR, antimicrobial resistance; ESBL+/NS, extended spectrum β‑lactamase-producing or not susceptible to ceftriaxone, cefotaxime, ceftazidime, or cefepime; FQ, fluoroquinolone; MDR-2/-3, multidrug-resistant if resistant to ≥ 1 antibiotic in ≥ 2 or ≥ 3 drug classes (including NTF, SXT, FQ, or the ESBL+/NS phenotype); NS, not susceptible; NTF, nitrofurantoin; SXT, trimethoprim/sulfamethoxazole; US, United States

**Supplementary Fig. 2** Observed NTF NS prevalence trends among 30-day non-duplicate *Klebsiella* spp*.* isolates from female outpatients in the US over the 2011–2019 study period


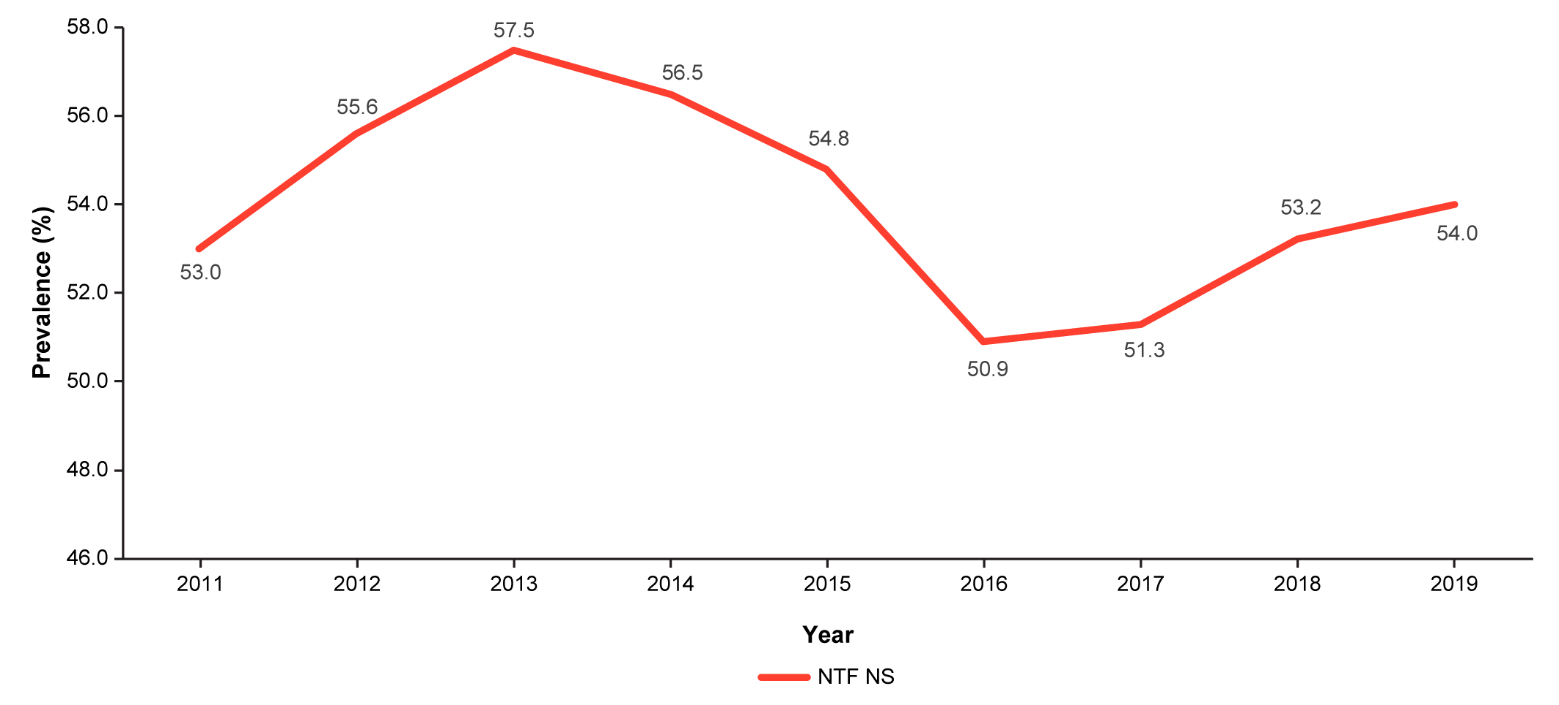


NS, not susceptible; NTF, nitrofurantoin; US, United States
